# Host Genetic Factors Associated with Symptomatic Primary HIV Infection and Disease Progression among Argentinean Seroconverters

**DOI:** 10.1371/journal.pone.0113146

**Published:** 2014-11-18

**Authors:** Romina Soledad Coloccini, Dario Dilernia, Yanina Ghiglione, Gabriela Turk, Natalia Laufer, Andrea Rubio, María Eugenia Socías, María Inés Figueroa, Omar Sued, Pedro Cahn, Horacio Salomón, Andrea Mangano, María Ángeles Pando

**Affiliations:** 1 Instituto de Investigaciones Biomédicas en Retrovirus y SIDA (INBIRS), Universidad de Buenos Aires-CONICET, Buenos Aires, Argentina; 2 Hospital Juan A. Fernandez, Buenos Aires, Argentina; 3 Fundación Huésped, Buenos Aires, Argentina; 4 Laboratorio de Biología Celular y Retrovirus, CONICET, Hospital de Pediatría “Prof. Dr. Juan P. Garrahan”, Buenos Aires, Argentina; South Texas Veterans Health Care System and University of Texas Health Science Center at San Antonio, United States of America

## Abstract

**Background:**

Variants in HIV-coreceptor C-C chemokine receptor type 5 (CCR5) and Human leukocyte antigen (HLA) genes are the most important host genetic factors associated with HIV infection and disease progression. Our aim was to analyze the association of these genetic factors in the presence of clinical symptoms during Primary HIV Infection (PHI) and disease progression within the first year.

**Methods:**

Seventy subjects diagnosed during PHI were studied (55 symptomatic and 15 asymptomatic). Viral load (VL) and CD4 T-cell count were evaluated. HIV progression was defined by presence of B or C events and/or CD4 T-cell counts <350 cell/mm^3^. CCR5 haplotypes were characterized by polymerase chain reaction and SDM-PCR-RFLP. HLA-I characterization was performed by Sequencing.

**Results:**

Symptoms during PHI were significantly associated with lower frequency of CCR5-CF1 (1.8% vs. 26.7%, p = 0.006). Rapid progression was significantly associated with higher frequency of CCR5-CF2 (16.7% vs. 0%, p = 0.024) and HLA-A*11 (16.7% vs. 1.2%, p = 0.003) and lower frequency of HLA-C*3 (2.8% vs. 17.5%, p = 0.035). Higher baseline VL was significantly associated with presence of HLA-A*11, HLA-A*24, and absence of HLA-A*31 and HLA-B*57. Higher 6-month VL was significantly associated with presence of CCR5-HHE, HLA-A*24, HLA-B*53, and absence of HLA-A*31 and CCR5-CF1. Lower baseline CD4 T-cell count was significantly associated with presence of HLA-A*24/*33, HLA-B*53, CCR5-CF2 and absence of HLA-A*01/*23 and CCR5-HHA. Lower 6-month CD4 T-cell count was associated with presence of HLA-A*24 and HLA-B*53, and absence of HLA-A*01 and HLA-B*07/*39. Moreover, lower 12-month CD4 T-cell count was significantly associated with presence of HLA-A*33, HLA-B*14, HLA-C*08, CCR5-CF2, and absence of HLA-B*07 and HLA-C*07.

**Conclusion:**

Several host factors were significantly associated with disease progression in PHI subjects. Most results agree with previous studies performed in other groups. However, some genetic factor associations are being described for the first time, highlighting the importance of genetic studies at a local level.

## Introduction

Research studies on primary HIV infection (PHI) are increasing worldwide to better understand the natural history of HIV infection and to identify the most important disease prognostic markers. As most of these studies were performed in other countries and due to genetic differences in the circulating virus and in the host, local studies are needed to better understand the particular characteristics of HIV infection dynamics [1].

In Argentina, an estimated 110,000 persons live with HIV (approximately 5,000 new cases per year) [2]. The first multicenter follow-up study of PHI (*Grupo Argentino de Seroconversión*) started in 2008. Retrospective and prospective data analyses allowed identifying factors associated with disease progression among untreated subjects. Symptomatic PHI, high VL (≥100,000 RNA copies/ml) or low CD4 T-cell count (≤350 cell/mm^3^) at baseline were identified as relevant factors for faster progression during the first year follow-up [3]. Data comparisons with other PHI cohorts revealed that VL at baseline in the Argentinean cohort was higher than those found in developed countries [4]–[5], closer to African and Asian levels [6]–[7]. Globally, 50–90% of subjects diagnosed during PHI are symptomatic [8]–[10], reaching 74% in the mentioned Argentinean cohort [3].

Previous studies demonstrated extensive variability in host susceptibility to HIV infection and disease progression [11]–[13]. Several host genetic factors affecting HIV infection and pathogenesis were identified, like chemokine receptors and HLA alleles [14]–[17]. Multiple variations were described in the CCR5 gene, in particular the 32 base-pair deletion (CCR5-Δ32). This deletion provides protection against HIV-1 infection with CCR5 tropic viruses in homozygotes and delays progression in heterozygous subjects [16], [18]–[19]. Seven Single Nucleotide Polymorphisms (SNPs) were defined in the *cis*-regulatory region between −2761 and −1835 of the CCR5 gene: −2733, −2554, −2459, −2135, −2132, −2086 and −1835 (GenBank accession number AF031236 and AF031237) [20]. Based on these variations and on the CCR2-V64I polymorphism, nine polymorphisms, called CCR5 Human Haplotypes (HH)-A, -B, -C, -D, -E, -F (F*1 and F*2), and –G (G*1 and G*2) were defined [15], [20]–[21]. One of the largest studies in the subject demonstrated that the frequency and effect of CCR5-HH differ among different ethnic groups. CCR5-HHA was associated with disease retardation among African-Americans, whereas CCR5-HHC did so among European-Americans. In the same study, specific sequences of CCR5-HHE were associated with higher transcriptional activity, surface expression and HIV/AIDS susceptibility [21]. Another factor associated with disease progression is the dose of the gene encoding CCL3L1 (MIP-1α), a natural ligand of CCR5. A previous study found an association between lower gene dose and disease progression, and this susceptibility is even greater in individuals with CCR5 genotypes associated with disease progression [22].

The HLA system has an impact on several aspects of HIV infection such as transmission, progression and therapeutic response [23]–[24]. HLA class I molecules are involved in peptide presentation to CD8 cytotoxic T lymphocytes (CTLs), which play a key role in reducing viral replication. HIV specific CD8 T-cell response emerges along with the control of viremia and resolution of clinical symptoms, which varies from person to person and constitutes a strong predictor of disease progression [25]–[26]. Heterozygosis at HLA class I region is considered to be a selective advantage because those individuals are able to present a greater range of antigenic peptides to CTLs than homozygotes, deferring the emergence of escape mutants and prolonging the period before the development of AIDS [18]. Even when several HLA alleles were associated with disease progression, HLA-B*27 and HLA-B*57 alleles showed a particularly strong association with delayed progression [27] and HLA-B*35 and HLA-B*53 with acceleration to AIDS [28].

Based on the effects of host genetic variations described on HIV disease progression, our aim was to analyze the association of CCR5/CCL3L1 system and HLA in the presence of clinical symptoms during PHI and disease progression within the first year post-infection.

## Materials and Methods

### Study population

A group of 70 individuals recruited through 2008–2012 was studied. Inclusion criteria for enrolment in the cohort were: >16 years old at first evaluation, PHI confirmed diagnosis, and first medical and laboratory evaluation (i.e., CD4 T-cell count and plasma HIV RNA) within six months of the probable date of infection. Primary HIV infection is defined as: (1) detection of HIV RNA or p24 antigen with a simultaneous negative or indeterminate Western blot assay [12]; or (2) positive Western blot with a negative test within the previous six months. Hence, it includes both acute and recently infected patients [3].

In this study, PHI was defined as symptomatic if one or more of the following symptoms, associated with acute retroviral syndrome, were present: fever, rash, lymphadenopathy, headache, oral ulcers, dysphagia or pharyngitis. Disease progression was defined by clinical B or C events (according to the Centers for Disease Control and Prevention 1993 classification [29]) and/or CD4 T-cell count <350 cells/mm^3^ within the first year of infection [3].

### Ethics Statement

International and national ethical guidelines for biomedical research involving human subjects were followed. This research study was reviewed and approved by a local Institutional Review Board (IRB), “*Fundación Huésped*” and was conducted in compliance with all federal regulations governing the protection of human subjects. All potential participants signed an informed consent prior to entering the study.

### Study Procedure

Once subjects were identified as PHI, they were included in the cohort. Subjects were evaluated at the time of diagnosis (baseline), at 6 months and at one year. On each visit, HIV plasma VL (branched-DNA, Versant HIV-1 RNA 3.0 assay, Siemmens Healthcare, USA), CD4 T-cell count (flow cytometry double platform, BD FACSCanto, BD Biosciences, USA), and clinical information were updated.

### Study samples

Peripheral blood samples were obtained on each visit. Whole blood samples or peripheral blood mononuclear cells (PBMC) were used for DNA extraction using QIAmp DNA Blood Mini Kit (QIAGEN GmbH, Hilden, Germany). Plasma samples from the first visit after HIV diagnosis were used for lipopolysaccharide (LPS) quantification (Limulus Amebocyte Lysate test, LAL assay, QCL-1000, Lonza, DK). HIV tropism was determined by sequencing a region of V3 loop from env gene (HXB2) [30]. Viral DNA was amplified in duplicate by nested PCR and amplicons were sequenced by Big Dye Terminator Kit (Amersham, Sweden). Viral tropism was inferred from Geno2Pheno algorithm (http://coreceptor.bioinf.mpi-inf.mpg.de/index.php) using a false positive rate of 10%.

### CCR5 and CCL3L1 characterization

CCR5-Δ32 deletion was identified by differences in PCR products size. CCR2 genotypes and Single Nucleotide Polymorphisms (SNPs) of the CCR5 gene corresponding to positions 29, 208, 627, 630, 676 and 927 (Genbank accession number: AF031236 and AF031237) [31] were determined with Site Directed Mutagenesis-PCR-Restriction Fragment Length Polymorphism (SDM-PCR-RFLP) assay. Primers used in each determination, PCR cycling condition and RFLP assay were reported previously [15], [21], [32]–[33]. Haplotype classification (HHA, HHB, HHC, HHD, HHE, HHF*1, HHF*2, HHG*1 and HHG*2) was determined as reported previously [15], [20]–[21]. CCL3L1 Copy Number (CN) was determined by Taqman real-time PCR [22].

### HLA characterization

HLA class I characterization was performed by sequencing-based typing (SBT). HLA-A exons 2 and 3 were amplified together. HLA-B and HLA-C exons 2 and 3 were amplified separately as reported in Table S1 and Figure S1 [34]–[36]. Amplicons were sequenced using the Big Dye Terminator sequencing kit (Amersham, Sweden) [36]. Sequence interpretation was performed using the NCBI SBT Interpretation software (http://www.ncbi.nlm.nih.gov/gv/mhc/sbt.cgi?cmd=main).

### Genetic score

Additive genetic score was used to compile host genetic information [37]. In our model, alleles with a previous reported protective effect were added, and risk alleles were subtracted. For CCR5 polymorphisms, Δ32 and CCR2-64I alleles were considered as protective (1) [21]. Regarding CCR5 genotypes, HHC/HHF*2 and HHC/HHG*2 were considered as protective (1), HHC/HHE, HHE/HHE and HHE/HHG*2 as deleterious (–1), and the others as neutral (0) [21], [32]. Two CCL3L1 cpg (mean in the Argentinean population) were considered as neutral (0). Lower CCL3L1 CN than the mean was considered as deleterious (–1) and higher CN as protective (1) [22]. HLA-A*02, HLA-A*32, HLA-A*68, HLA-B*15, HLA-B*13, HLA-B*27, HLA-B*32, HLA-B*39, HLA-B*44, HLA-B*51 and HLA-B*57 were considered as protective (1). HLA-A*11, HLA-A*23, HLA-A*24, HLA-B*08, HLA-B*35, HLA-B*53, HLA-C*04 and HLA-C*07 were considered as deleterious (–1). Other HLA alleles were considered as neutral (0) [11]–[13], [23]–[24], [27]–[28], [37]–[39]. Heterozygosis for HLA was considered as protective (1) and homozygosis as deleterious (–1) [18].

### Statistical analysis

Baseline characteristics were described using mean or medians and standard deviation or interquartile ranges [IQRs] for continuous variables respectively, and counts and percentages for categorical data. Chi-square test or Fisher’s exact test were used to compare proportions. Differences among continuous variables were analyzed using Student’s t-test or Wilcoxon test. Spearman correlation was calculated for genetic score and HIV viral load and CD4 T-cell count (baseline and follow up). All p values were two-sided; p values<0.05 were considered to be statistically significant. Lack of complete data values in table is expressed in numbers. Data analysis was performed using SPSS 15.0, 2007 (Chicago, Illinois).

## Results

### Characteristics of the study population

We studied 70 HIV-infected adults diagnosed during primary HIV infection (PHI) (49 men and 21 women), 55 were symptomatic and 15 asymptomatic. Sixty of them were also classified according to disease progression within the first year post diagnosis, 18 progressed and 42 did not. Most PHI subjects were recruited during Fiebig stages V and VI [40]. Sexual transmission was reported as the main route: all the women reported heterosexual transmission whereas 82.2% of men reported sexual relationship with other men as the probably route of acquisition of the virus. All subjects were from Buenos Aires City and surrounding areas. The population of this area is mostly descendent from South Europe [41]. Median HIV VL at diagnosis was 61862 RNA copies/ml, whit significantly higher VL in those who presented symptoms and those who progressed (Table 1). The same trend was observed for VL at 6 months. Baseline CD4 T-cell count was 514 cells/mm^3^ without statistical differences between symptomatic and asymptomatic subjects. Significantly higher CD4 T-cell counts (baseline, 6 and 12 months) were observed among subjects who did not progress to disease during the first year (Table 1).

**Table 1:** HIV viral load and CD4 T-cell count of the study population diagnosed during primary HIV infection [PHI] (N = 70).

		Symptomatic PHI		p	Progressor at one year		p	All (N = 70)
		Yes (N = 55)	No (N = 15)		Yes (N = 18)	No (N = 42)		
HIV RNAmediancopies/ml(IQR)	Baseline	77,080	7,024	**0.003**	193,601	41,402	**0.003**	61,862
		(30,449–386,715)	(2,699–76,466)		(80,545–500,000)	(10,409–154,476)		(17,050–257,524)
	6 month	66,002	9,018	**0.004**	166,812	33,508	**0.001**	40,231
		(17.959–178.030)	(3,820–34,624)		(47,167–321,018)	(8,578–73,231)		(117,17–165,238)
CD4 T-cellcountmediancells/mm^3^(IQR)	Baseline	502	587	0.322	306	602	**<0.001**	514
		(356–649)	(416–876)		(237–346)	(500–741)		(387–671)
	6 month	499	555	0.694	323	602	**<0.001**	503
		(356–665)	(424–665)		(172–386)	(488–690)		(404–65)
	12 month	491	534	0.296	330	534	**0.001**	501
		(389–615)	(436–672)		(289–504)	(435–643)		(400–619)

PHI: primary HIV infection. IQR: interquartile range. Statistically significant p values are in bold.

### Frequency of CCR5 haplotypes/genotypes and CCL3L1

**Figure 1:**
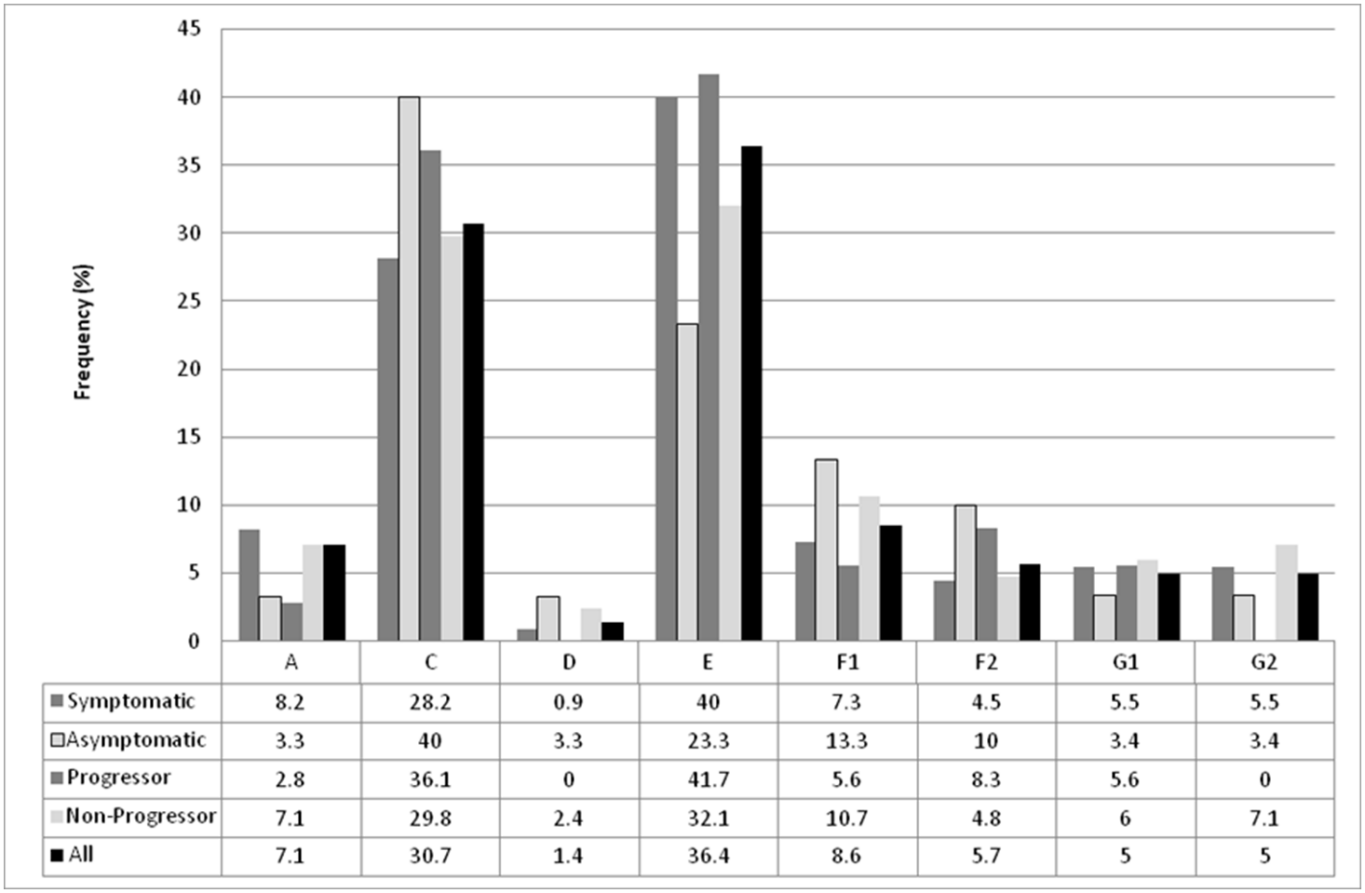
Frequency of CCR5 haplotypes of the study population diagnosed during primary HIV infection [PHI] (N = 70). Full information is available on supplementary material (Table S1).

Similar to the results found in Argentinean children exposed perinatally to HIV (including both HIV infected and not infected) [42] and blood donors [43], the most frequent CCR5 haplotypes in the PHI group were HHE (36.4%) and HHC (30.7%). Frequencies of all the other haplotypes were lower than 10% (Figure 1; Table S2). Regarding CCR5 genotypes, HHC/HHE (21.4%) and HHE/HHE (12.9%) were the most commonly found. Other genotypes were present with frequencies lower than 10% (Table 2 and Table S3). The CCL3L1 gene copy number, one of the main ligands of CCR5, was evaluated in 50 PHI subjects with a median of two copies (IQR25-75, 1–4), as reported in persons of European origin [22].

**Table 2:** Frequency of CCR5 human genotypes of the study population diagnosed during primary HIV infection [PHI] (N = 70).

Genotype	Symptomatic PHI		p	Progressor at one year		p	All (N = 70)
	Yes (N = 55)*	No (N = 15)*		Yes (N = 18)*	No (N = 42)*		
HHC/HHC	2 (3.6)	1 (6.7)	0.521	2 (11.1)	1 (2.4)	0.212	3 (4.3)
HHC/HHE	12 (21.8)	3 (20)	1.000	4 (22.2)	9 (21.4)	1.000	15 (21.4)
**HHC/HHF** * **1**	**1 (1.8)**	**4 (26.7)**	**0.006**	1 (5.6)	4 (9.5)	1.000	5 (7.1)
**HHC/HHF** * **2**	3 (5.5)	1 (6.7)	1.000	**3 (16.7)**	**0**	**0.024**	4 (5.7)
HHC/HHG*1	5 (9.1)	1 (6.7)	1.000	1 (5.6)	5 (11.9)	0.658	6 (8.6)
HHE/HHE	8 (14.5)	1 (6.7)	0.672	4 (22.2)	3 (7.1)	0.220	9 (12.9)
HHE/HHF*1	6 (10.9)	0	0.329	1 (5.6)	4 (9.5)	1.000	6 (8.6)

*Data are no. (%) of CCR5 haplotypes.

### Frequency of HLA variants

Given the essential role of CTL responses during PHI as well as the description of a strong association among certain HLA-I alleles with virus control, HLA-I frequencies were studied in this cohort finding 17 HLA-A, 27 HLA-B and 14 HLA-C different alleles. The HLA-A alleles most frequently found were HLA-A*02 (27.2%) and HLA-A*24 (12.5%). In HLA-B locus, HLA-B*35 (15.6%) and HLA-B*44 (12.9%) were the most frequent. In HLA-C, HLA-C*07 (27.9%), HLA-C*04 (16.2%) and HLA-C*03 (11.8%) were the most frequent. Other HLA-A, B and C alleles showed frequencies lower than 10% (Table S4). HLA class I alleles were found in homozygosis in the following frequencies: 32.4% for HLA-A, 3.0% for HLA-B and 17.6% for HLA-C (Table S5). The most common combinations for HLA-A were A*02-A*02 (11.8%) and A*02-A*68 (8.8%), for HLA-B were B*15-B*35 (4.5%) and B*35-B*44 (4.5%), and for HLA-C, C*04-C*07 (8.8%), C*07-C*07 (8.8%) and C*03-C*07 (7.4%) (data not shown).

### Influence of CCR5 haplotypes/genotypes, CCL3L1 copy number, and HLA variants on symptoms present during acute HIV infection

In order to identify individual host genetic determinants of early HIV disease progression, the PHI cohort was stratified according to the presence/absence of symptoms during the seroconversion period. Regarding the CCR5 coreceptor, HHC was overrepresented (40% vs. 28.2%) and HHE (23.3% vs. 40%) was less frequent in asymptomatic as compared to symptomatic subjects, however without statistical significance (Figure 1). Concerning CCR5 genotypes, HHC/HHF*1 was detected in a significantly higher percentage among asymptomatic subjects (26.7% vs. 1.8%, p = 0.006). Even when it was not statistically significant, genotype HHE/HHF*1 was only found among symptomatic subjects (10.9%) (Table 2 and Table S3). No significant differences were found in the CCL3L1 copy number, even when a higher copy number was detected among asymptomatic (median (IQR25-75); 3 (2–3) and 2 (1–4), respectively). No influence of HLA-A, -B and -C alleles was detected in the presence of symptoms during PHI (Table S4). Likewise, no influence of HLA homozygosis was observed in the presence of symptoms during seroconversion (Table S5). When HLA pairs were compared, HLA-B*35-B*44 was found in a significantly higher frequency among asymptomatic subjects (21.4% vs. 0%, p = 0.007) (data not shown).

Only CCR5 genotypes with a frequency higher than 10% in some of the study groups were included in the table. No significant differences were observed among CCR5 genotypes with frequencies lower than 10%. Full information is on supplementary material (Table S2).

### Influence of CCR5 haplotypes/genotypes, CCL3L1 and HLA variants on disease progression within the first year

Additionally, the PHI group was analyzed in order to identify possible genetic factors that might influence the rate of progression within the first year. Several CCR5 haplotypes were most frequently detected in individuals who did not progress (e.g. HHA, HHF*1 and HHG*2) and HHF*2 was most represented in subjects who progressed to disease (Table S2), without statistical differences. Regarding CCR5 genotypes, HHC/HHF*2 was significantly associated with progression (p = 0.024) and a higher, but not significant proportion of subject who progress had HHE/HHE also as compared with those who do not progress (22.2% vs. 7.1%) (Table S3). Regarding HLA alleles, a strong association was found between disease progression and higher frequency of HLA-A*11 (16.7% vs. 1.2%, p = 0.003) and lower frequency of HLA-C*03 (17.5% vs. 2.8%, p = 0.035) (Table S4). No influence of HLA homozygosis was observed in disease progression (Table S5).

### Influence of CCR5 haplotypes/genotypes, CCL3L1 and HLA variants on plasma HIV viral load and CD4 T-cell count

As the CD4 T-cell count and HIV plasma VL are good predictors of disease progression [3], the association of these parameters with host genetic factors was also analyzed. Subjects with CCR5 HHE haplotype had higher VL after 6 months (66,001 copies/ml vs. 31,718 copies/ml, p = 0.039) and also higher baseline VL (98,684 copies/ml vs. 41,402 copies/ml, p = 0.082). On the other hand, HHA was found to be associated with higher baseline CD4 T-cell levels (656 cells/mm^3^ vs. 499 cells/mm^3^, p = 0.044). Regarding CCR5 genotypes, HHC/HHF*1 was associated with lower VL (6,243 copies/ml vs. 53,997 copies/ml, p = 0.027) and HHC/HHF*2 with lower CD4 T-cell levels at baseline (379 cells/mm^3^ vs. 545 cells/mm^3^, p = 0.046), at 6 months (355 cells/mm^3^ vs. 531 cells/mm^3^, p = 0.024) and at 12 months (290 cells/mm^3^ vs. 510 cells/mm^3^, p = 0.034).

Concerning the HLA influence on CD4 T-cell count and HIV plasma VL, the presence of several alleles was found to be beneficial for HIV subjects, with an association with higher CD4 T-cell count (HLA-A*01, HLA-A*23, HLA-B*07, HLA-B*39 and HLA-C*07) or lower HIV plasma VL (HLA-A*31 and HLA-B*57). Conversely, some alleles were found to be detrimental for subjects, with an association with higher HIV plasma VL (HLA-A*11, HLA-A*24 and HLA-B*53) or lower CD4 T-cell count (HLA-A*24, HLA-A*33, HLA-B*14, HLA-B*53 and HLA-C*08) (Table 3).

**Table 3:** HIV viral load and CD4 T-cell count of the study population diagnosed during primary HIV infection [PHI] according to HLA alleles (N = 70).

Alleles		CD4 T-cell count			HIV RNA	
		median cells/mm^3^			median copies/ml	
		Baseline	6 months	12 months	Baseline	6 months
HLA-A*01	Yes	902	810	716	5160	4298
	No	500	499	491	64045	40083
	p	**0.022**	**0.019**	0.112	0.241	0.317
HLA-A*11	Yes	347	344	475	477708	166930
	No	525	517	492	52352	38270
	p	0.070	0.071	0.447	**0.020**	0.059
HLA-A*23	Yes	736	637	534	36101	24322
	No	499	497	475	61862	40232
	p	**0.038**	0.072	0.195	0.374	0.290
HLA-A*24	Yes	393	403	483	500000	89517
	No	576	545	500	41402	30591
	p	**0.049**	**0.048**	0.371	**0.001**	**0.004**
HLA-A*31	Yes	602	563	612	24654	19603
	No	502	502	491	67397	56594
	p	0.494	0.616	0.883	**0.032**	**0.038**
HLA-A*33	Yes	387	387	347	67660	67660
	No	535	528	515	55276	39484
	p	**0.046**	0.100	**0.021**	0.818	1.00
HLA-B*07	Yes	535	818	679	378025	133268
	No	525	499	474	52352	38720
	p	0.972	**0.015**	**0.005**	0.177	0.280
HLA-B*14	Yes	466	485	410	213099	117061
	No	575	542	534	52352	37506
	p	0.167	0.135	**0.002**	0.229	0.260
HLA-B*39	Yes	644	780	573	4383	18062
	No	509	497	483	62679	42753
	p	0.098	**0.027**	0.175	0.073	0.085
HLA-B*53	Yes	288	248	286	500000	349244
	No	545	531	509	54286	39033
	p	**0.046**	**0.036**	0.117	0.058	**0.028**
HLA-B*57	Yes	525	495	654	16926	12971
	No	529	528	492	66821	47077
	p	0.819	0.865	0.272	**0.046**	0.058
HLA-C*07	Yes	525	527	534	66821	60546
	No	497	491	449	62679	40083
	p	0.738	0.527	**0.038**	0.584	0.563
HLA-C*08	Yes	437	499	409	184000	163664
	No	519	499	533	61045	40083
	p	0.290	0.200	**0.001**	0.443	0.286

### Additive genetic score

**Figure 2:**
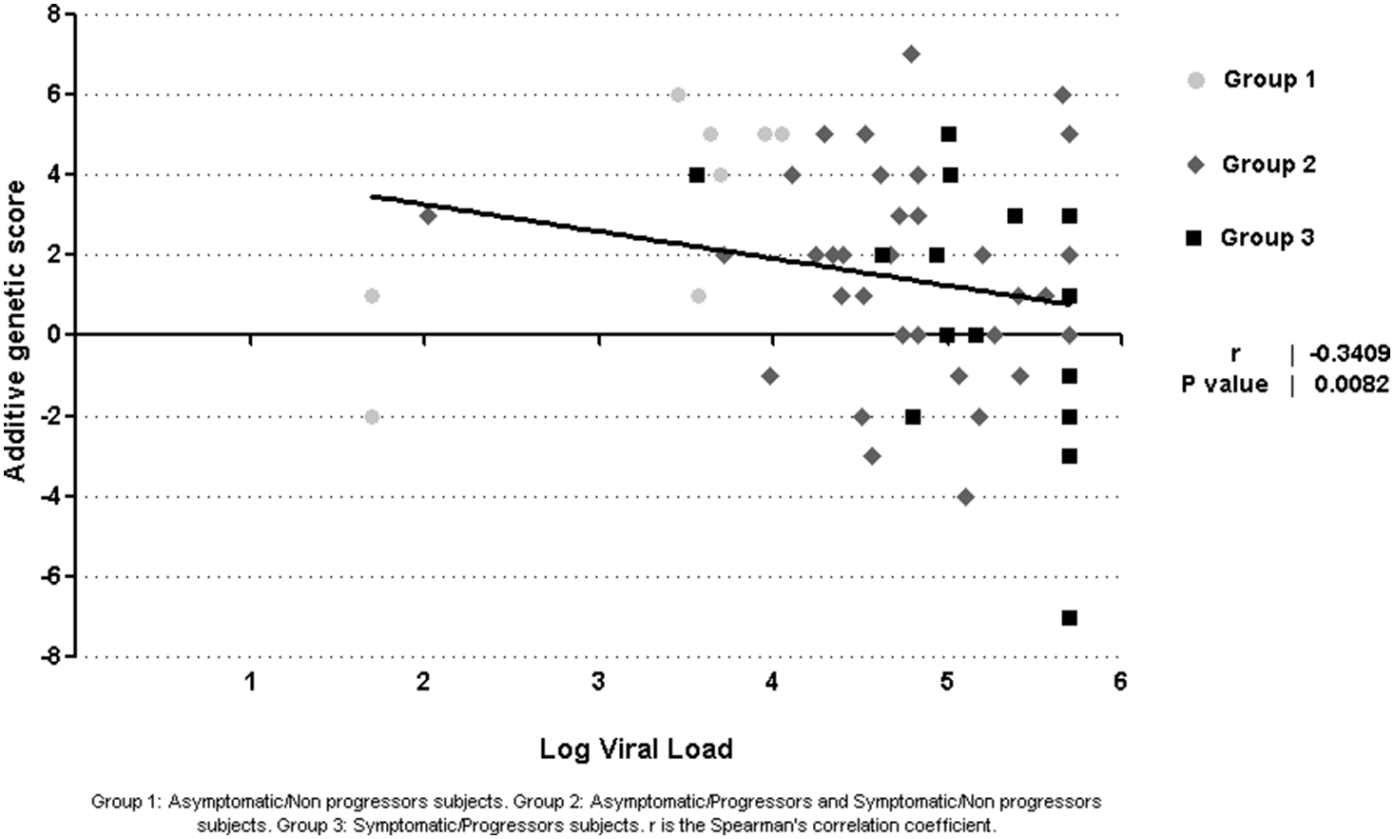
Correlation between baseline HIV viral load and additive genetic score on the study population diagnosed during primary HIV infection [PHI] (N = 70).

Additive genetic score was calculated for each subject and average values were calculated considering symptoms during PHI (2.6 for asymptomatic and 1.4 for symptomatic subjects) and disease progression within the first year (1.8 for those who did not progress and 0.6 for those who progressed). Subjects were grouped according to both characteristics: Group 1: Asymptomatic/Non-progressors, Group 2: Asymptomatic/Progressors and Symptomatic/Non-progressors, and Group 3: Symptomatic/Progressors. Mean genetic score was: 2.8, 1.6 and 0.5 for groups 1, 2 and 3, respectively. Correlation analyses revealed a significant negative correlation between genetic score and HIV viral load at baseline (p = 0.008) (Figure 2). No significant association was observed between genetic score and CD4 T-cells count.

### Complementary studies

HIV infection has been associated with disruption of mucosal barrier and CD4 T-cell depletion in the gastrointestinal tract. This damage is caused, at least in part, by increased translocation of microbial products, mainly lipopolysaccharides (LPS), a major component of gram-negative bacterial cell walls [44]–[46]. Since immune activation is a good predictor of disease progression, plasma LPS levels were determined in the baseline sample of 65 individuals finding a median of 39.0 pg/ml (IQR25-75, 26.7–56.8) with significantly higher levels in the symptomatic than the asymptomatic group (43.5 pg/ml vs. 29.0 pg/ml, p = 0.040). No association was found among LPS levels, disease progression, CD4 T-cell count, HIV VL or host genetic factors. HIV tropism was determined given that the presence of X4 tropic viruses was associated with a more rapid disease progression (data not shown). Fourteen out of 59 (23.7%) PHI subjects presented X4 tropic HIV variants. Even when no statistically significant differences were observed, X4 tropic HIV variants were overrepresented among symptomatic subjects (26.1% vs. 15.4%, p = 0.713). No differences were observed among HIV tropism, disease progression, CD4 T-cell count or HIV VL.

## Discussion

Other countries reported associations between human genes and HIV susceptibility. However, local studies are needed considering differences in genetic background [14], [17], [19]. In line with this, for the first time in Argentina, this study reports several human genes associated with early HIV disease progression among adults.

Buenos Aires population is mainly descendant of Southern Europe. The frequency of CCR5 haplotypes reported here correlates with reports in Hispanic and other Argentinean groups [21], [43], with HHE and HHC being the most common haplotypes. Regarding CCR5 genotypes, the most common were HHC/HHE and HHE/HHE, with other genotypes having frequencies lower than 10%. In comparison with blood donors, PHI individuals were found to have a higher but not significant frequency of HHE/HHE genotype (5.9% vs. 12.9% respectively). This result is consistent with previous reports evidencing an association between presence of HHE/HHE genotype and enhancement of HIV infection [21], [42]. Even when the HHE haplotype and the HHE/HHE genotype were overrepresented among symptomatic subjects and those who progressed, no significant associations were observed, maybe due to sample size. Data on HIV VL also supports the same trend with significantly higher VL at 6 months among subjects carrying HHE. This trend is in line with previous studies that associated disease progression with HHE [21], [42]. However, this disease-modified effect was not observed among other ethnic groups (i.e., Africans) where the frequency of HHE haplotype was much lower (≈18%) [21]. As HHE is the most frequent CCR5 haplotype in our cohort, the potential adverse effect of this haplotype deserves special attention.

HHC/HHF*1 genotype was associated with asymptomatic PHI and HHC/HHF*2 with disease progression. In line with these results, we found that the HHC/HHF*1 genotype was associated with lower levels of VL and HHC/HHF*2, with lower CD4 T-cell levels at baseline and during one-year follow-up. Only few studies support these findings, maybe due to the fact that these genotypes were found in low frequency in most cohorts [21], [42]. One of the most important studies in the subject found a disease accelerating effect for HHC/HHF*1 among African Americans [21]. However, this study also reports that the effect of HHC haplotypes on HIV disease differed among ethnic groups. While the HHC haplotype in African Americans was associated with disease acceleration, in Caucasians and Hispanics it was associated with disease retardation. Regarding the HHF*2 haplotype, a previous report found similar results in individuals carrying the allele with lower CD4 T-cell counts during follow-up [47]. However, these results disagree with previous studies that observed a protective effect against disease progression among subjects carrying the CCR2-64I allele [33]. HHC/HHF*2 genotype was also associated with disease retardation among Argentinean children [42]. Even when no statistically significant association was established, the CCR5 genotype HHE/HHF*1 was only detected among symptomatic subjects in more than 10% of the group. CCL3L1 copy number distribution in PHI population was similar to that observed in the European population [22] with a median of two copies. Even when no significant differences were observed, asymptomatic individuals had a higher copy number, maybe suggesting that CCL3L1 would have an impact since the HIV infection onset.

Identifying HLA alleles associations with HIV disease progression is complex due to the extreme variability of the loci. In fact, this study identifies 17, 27 and 14 HLA-A, B and C alleles, respectively. Coincident with previous reports, including our blood donors group, the alleles most frequently reported here were HLA-A*02 and HLA-*24, HLA-B*35 and HLA-B*44, and HLA-C*07, HLA-C*04 and HLA-C*03 [41]. Even when it was proposed that heterozygosis on HLA confers advantages on disease progression revealing a greater variety of the immune response [18], [48]–[49], no significant differences in disease progression were detected between heterozygotes and homozygotes at any individual HLA locus or homozygosis at one, two, or all three class I loci.

Several HLA alleles identified in our study were associated with disease progression. Our results adds more evidence to the protective effect of HLA-B*57 allele on disease progression [23], with significantly lower VL at baseline and also lower, but not significant, VL at 6 months. Moreover, the allele was only found among those who did not progress. Even when HLA-B*57 was previously associated with the absence of symptoms during seroconversion, our study failed to confirm these findings [50]. Regarding HLA-B*27, reported as a protective allele [50], we did not observe this trend or evidence, likely due to the low frequency found (1.5% among HIV positive and 2.0% among blood donors). Another HLA allele, several times associated with disease progression is HLA-B*35 [18], [23]. However, our study did not find any statistical association or trend even when the frequency of the allele was around 15% in the overall group.

HLA-A*11 was associated with disease progression during the first year and with higher VL at baseline. We also found a trend in the presence of the allele and higher HIV VL at 6 months and lower CD4 T-cell counts at baseline and during follow-up. These results agree with a previous study that found a higher frequency of HLA-A*1101 among subjects with AIDS compared with other HIV subjects who did not progress [51]. Even when this study performed high resolution HLA-typing, in contrast to our low resolution data, it is important to mention that typing studies reported that most of the typed HLA-A*11 are HLA-A*1101 [41], [52].

HLA-B*53 was associated with lower CD4 T-cell counts and higher HIV VL levels, even when only two subjects carried that allele. Elevated VL levels among subjects with HLA-B*53 were previously observed among African seroconverters [53]. Although only few subjects carried the HLA-B*53, the potential impact of this allele on disease progression may deserve more investigation. Another interesting allele is HLA-A*24, associated with lower CD4 T-cell counts and higher VL levels at baseline and during follow-up. This allele frequency was also higher (but not significant) among subjects who presented symptoms during seroconversion as compared with those without them. Previous studies also found a deleterious effect of this allele, enhancing HIV infection [54], showing rapid decline in CD4 T-cells [27] and favouring disease progression [55]. HLA-B*39 confers a beneficial effect on disease evolution yielding high CD4 T-cell counts and low VL levels [16], [55]. We also observed a trend in higher frequency of HLA-B*39 among asymptomatic vs. symptomatic (10.7% vs. 2.9%) subjects. Controversial results were found in other alleles. While our study suggests that subjects with HLA-B*14 (with significantly lower CD4 T-cell counts at 12 months and a trend of lower levels of CD4 T-cells at baseline and at 6 months and higher VL) progressed faster to disease, others found significant associations between allele and low disease progression [56] and that the allele had enhanced HIV infection [57].

Previous studies showed that plasma LPS levels among subjects with acute HIV infection were similar to non-infected subjects [58]. In fact, our study found similar levels in the PHI group (39.0 pg/ml) and a group of HIV-negative subjects (37.4 pg/ml, data not shown). However, we found that higher plasma LPS levels are significantly associated with presence of symptoms during PHI. These results suggest higher immune activation in symptomatic subjects since the establishment of infection.

An important limitation of the current research was the low frequency of asymptomatic subjects included due to the difficulty in identifying them during the seroconversion period. The lack of progression data in a group of patients also influenced the possibility of finding significant associations. It is also important to note the difficulty in finding associations when genetic variants are in low frequency. Given these limitations, a score was constructed in order to combine some of the most important human genetic factors previously associated with HIV/AIDS and to look for associations with presence of symptoms, disease progression and other progression markers like HIV viral load and CD4 T-cell count. Results reveal a higher score in asymptomatic and those who did not progress, revealing the presence of more protective genetic factors in these groups. Even more, when data were analysed considering both variables (symptoms and progression) a higher score was observed for those who did not present symptoms during PHI and did not progress at one year. As described by other authors, the genetic score was a useful tool to evaluate the additive influence of human genetic factors with high variability on small groups [37].

## Conclusions

This study reveals that some host genetic variants identified previously as disease-modificating factors influence disease progression from the very beginning of the HIV infection. However, here we also described some associations for the first time. Variability of host genetic factors as well as their association with HIV infection and/or disease progression relies strongly on the ethnic population background. Therefore, the population ethnicities are growing it is becoming increasingly difficult to extrapolate results from one study to other populations. In this context, it is important to highlight the need to perform studies at a in this setting not only these genetic differences in the population but also the environmental variance and the circulating virus.

## Supporting Information

Figure S1 **PCR Cycle conditions for HLA class I characterization.**
(DOC)

Table S1 **Sequences of primers used for HLA class I characterization.**
(DOC)

Table S2 **Frequency of CCR5 haplotypes of the study population diagnosed during primary HIV infection [PHI] (N = 70).**
(DOC)

Table S3 **Frequency of CCR5 human genotypes among the study population diagnosed during primary HIV infection [PHI] (N = 70).**
(DOC)

Table S4 **Frequency of HLA class I alleles among the study population diagnosed during primary HIV infection [PHI].**
(DOC)

Table S5 **Frequency of HLA class I alleles homozygosis among the study population diagnosed during primary HIV infection [PHI].**
(DOC)
