# Supplementary material for: Host Genetic Factors Associated with Symptomatic Primary HIV Infection and Disease Progression among Argentinean Seroconverters
Source: PLoS One. 2014 Nov 18;9(11):e113146. doi: 10.1371/journal.pone.0113146 (PMC4236131; doi:10.1371/journal.pone.0113146)
Supplement: Figure S1 — PCR Cycle conditions for HLA class I characterization. (DOC) [file pone.0113146.s001.doc]

**Figure S1**. PCR Cycle conditions
