# Supplementary material for: Host Genetic Factors Associated with Symptomatic Primary HIV Infection and Disease Progression among Argentinean Seroconverters
Source: PLoS One. 2014 Nov 18;9(11):e113146. doi: 10.1371/journal.pone.0113146 (PMC4236131; doi:10.1371/journal.pone.0113146)
Supplement: Table S1 — Sequences of primers used for HLA class I characterization. (DOC) [file pone.0113146.s002.doc]

**Table S1**.Sequences of primers used for HLA class I characterization.

| Gene | | PCR primers | | |  | | Sequencing primers | | | |
| --- | --- | --- | --- | --- | --- | --- | --- | --- | --- | --- |
|  |  | Name | Sequence | |  | | Name | | Sequence | |
| HLA-A | Exons 2 and 3 | 5AIn1-46 | 5’ GAAACSGCCTCTGYGGGGAGAAGCAA 3’ | |  | | 3AIn2-37 | | 5’ GGCCCGTCCGTGGGGGATGAG 3’ | |
|  |  | 3AIn3-66 | 5’ TGTTGGTCCCAATTGTCTCCCCTC 3’ | |  | | 3AIn3-41 | | 5’ TGTGGGAGGCCAGCCCGGGAGA 3’ | |
| HLA-B | Exon 2 | 5BE2.2b | 5’ GGGAGGAGCGAGGGGACCGCAG 3’ | |  | | B44 | | 5’ GGATGGGGAGTCGTGACCT 3’ | |
|  |  | B44 | 5’ GGATGGGGAGTCGTGACCT 3’ | |  | |  |  |  |  |
|  | Exon 3 | 5BInt2 | 5’ ACKGKGCTGACCGCGGGG 3’ | |  | | 3B1 | | 5’ CCATCCCCGGCGACCTATAGGAGATG 3’ | |
|  |  | 3BIn3-37 | 5’ GGAGGCCATCCCCGGCGACCTAT 3’ | |  | |  |  |  |  |
|  |  | 3B1 | 5’ CCATCCCCGGCGACCTATAGGAGATG 3’ | |  | |  |  |  |  |
| HLA-C | Exon 2 | 5CIn1-61 | 5’ AGCGAGGKGCCCGCCCGGCGA 3’ | |  | | 5CIn1-77 | | 5’ GGAGCCGCGCAGGGAGGWGGGTC 3’ | |
|  |  | 5CIn1-77 | 5’ GGAGCCGCGCAGGGAGGWGGGTC 3’ | |  | |  |  |  | |
|  |  | 3C26 | 5’ GGAGGGGTCGTGACCTGCGC 3’ | |  | | 3C26 | | 5’ GGAGGGGTCGTGACCTGCGC 3’ | |
|  | Exon 3 | 5CInt2 | 5’ CTTGACCRCGGGGGGCGGGG 3’ | |  | | 5CInt2 | | 5’ CTTGACCRCGGGGGGCGGGG 3’ | |
|  |  | 3BCIn3-12 | | 5’ GGAGATGGGGAAGGCTCCCCACT 3’ | |  | | 3BCIn3-12 | | 5’ GGAGATGGGGAAGGCTCCCCACT 3’ |
