# Supplementary material for: Host Genetic Factors Associated with Symptomatic Primary HIV Infection and Disease Progression among Argentinean Seroconverters
Source: PLoS One. 2014 Nov 18;9(11):e113146. doi: 10.1371/journal.pone.0113146 (PMC4236131; doi:10.1371/journal.pone.0113146)
Supplement: Table S2 — Frequency of CCR5 haplotypes of the study population diagnosed during primary HIV infection [PHI] (N = 70). (DOC) [file pone.0113146.s003.doc]

**Table S2**. Frequency of CCR5 haplotypes of the study population diagnosed during primary HIV infection [PHI] (N=70).

| HH | Symptomatic PHI | |  | Progressor at one year | |  | All (N=70)* |
| --- | --- | --- | --- | --- | --- | --- | --- |
|  | Yes (N=55)* | No (N=15)* | p | Yes (N=18)* | No (N=42)* | p |  |
| HHA | 9 (8.2) | 1 (3.3) | 0.689 | 1 (2.8) | 6 (7.1) | 0.673 | 10 (7.1) |
| HHC | 31(28.2) | 12(40) | 0.265 | 13 (36.1) | 25 (29.8) | 0.525 | 43 (30.7) |
| HHD | 1 (0.9) | 1 (3.3) | 0.384 | 0 | 2 (2.4) | 1.000 | 2 (1.4) |
| HHE | 44 (40) | 7(23.3) | 0.133 | 15 (41.7) | 27 (32.1) | 0.404 | 51 (36.4) |
| HHF*1 | 8 (7.3) | 4 (13.3) | 0.286 | 2 (5.6) | 9 (10.7) | 0.502 | 12 (8.6) |
| HHF*2 | 5 (4.5) | 3 (10) | 0.368 | 3 (8.3) | 4 (4.8) | 0.427 | 8 (5.7) |
| HHG*1 | 6 (5.5) | 1 (3.4) | 1.000 | 2 (5.6) | 5 (6.0) | 1.000 | 7 (5.0) |
| HHG*2 | 6 (5.5) | 1 (3.4) | 1.000 | 0 | 6 (7.1) | 0.165 | 7 (5.0) |

*Data are no. (%) of CCR5 haplotypes.
