# Supplementary material for: Host Genetic Factors Associated with Symptomatic Primary HIV Infection and Disease Progression among Argentinean Seroconverters
Source: PLoS One. 2014 Nov 18;9(11):e113146. doi: 10.1371/journal.pone.0113146 (PMC4236131; doi:10.1371/journal.pone.0113146)
Supplement: Table S3 — Frequency of CCR5 human genotypes among the study population diagnosed during primary HIV infection [PHI] (N = 70). (DOC) [file pone.0113146.s004.doc]

**Table S3.** Frequency of CCR5 human genotypes among the study population diagnosed during primary HIV infection [PHI] (N=70).

| Genotype | Symptomatic PHI | | p | Progressor at one year | | p | All (N=70) |
| --- | --- | --- | --- | --- | --- | --- | --- |
|  | Yes (N=55) | No (N=15) |  | Yes (N=18) | No (N=42) |  |  |
| HHA/HHC | 3 (5.5) | 0 | 1.000 | 0 | 1 (2.4) | 1.000 | 3 (4.3) |
| HHA/HHE | 5 (9.1) | 1 (6.7) | 1.000 | 1 (5.6) | 4 (9.5) | 1.000 | 6 (8.6) |
| HHA/HHF*1 | 1 (1.8) | 0 | 1.000 | 0 | 1 (2.4) | 1.000 | 1 (1.4) |
| HHC/HHC | 2 (3.6) | 1 (6.7) | 0.521 | 2 (11.1) | 1 (2.4) | 0.212 | 3 (4.3) |
| HHC/HHD | 0 | 1 (6.7) | 0.214 | 0 | 1 (2.4) | 1.000 | 1 (1.4) |
| HHC/HHE | 12 (21.8) | 3 (20) | 1.000 | 4 (22.2) | 9 (21.4) | 1.000 | 15 (21.4) |
| **HHC/HHF*1** | **1 (1.8)** | **4 (26.7)** | **0.006** | 1 (5.6) | 4 (9.5) | 1.000 | 5 (7.1) |
| **HHC/HHF*2** | 3 (5.5) | 1 (6.7) | 1.000 | **3 (16.7)** | **0** | **0.024** | 4 (5.7) |
| HHC/HHG*1 | 5 (9.1) | 1(6.7) | 1.000 | 1 (5.6) | 5 (11.9) | 0.658 | 6 (8.6) |
| HHC/HHG*2 | 3 (5.5) | 0 | 1.000 | 0 | 3 (7.1) | 0.547 | 3 (4.3) |
| HHD/HHE | 1 (1.8) | 0 | 1.000 | 0 | 1 (2.4) | 1.000 | 1 (1.4) |
| HHE/HHE | 8 (14.5) | 1 (6.7) | 0.672 | 4 (22.2) | 3 (7.1) | 0.220 | 9 (12.9) |
| HHE/HHF*1 | 6 (10.9) | 0 | 0.329 | 1 (5.6) | 4 (9.5) | 1.000 | 6 (8.6) |
| HHE/HHF*2 | 1 (1.8) | 0 | 1.000 | 0 | 1 (2.4) | 0.300 | 1 (1.4) |
| HHE/HHG*1 | 1 (1.8) | 0 | 1.000 | 1 (5.6) | 0 | 1.000 | 1 (1.4) |
| HHE/HHG*2 | 2 (3.6) | 1 (6.7) | 0.521 | 0 | 2 (4.8) | 1.000 | 3 (4.3) |
| HHF*2/HHF*2 | 0 | 1 (6.7) | 0.214 | 0 | 1 (2.4) | 1.000 | 1 (1.4) |
| HHG*2/HHF*2 | 1 (1.8) | 0 | 1.000 | 0 | 1 (2.4) | 1.000 | 1 (1.4) |

*Data are no. (%) of patients having the CCR5 genotype.
