# Supplementary material for: Host Genetic Factors Associated with Symptomatic Primary HIV Infection and Disease Progression among Argentinean Seroconverters
Source: PLoS One. 2014 Nov 18;9(11):e113146. doi: 10.1371/journal.pone.0113146 (PMC4236131; doi:10.1371/journal.pone.0113146)
Supplement: Table S4 — Frequency of HLA class I alleles among the study population diagnosed during primary HIV infection [PHI]. (DOC) [file pone.0113146.s005.doc]

**Table S4.** Frequency of HLA class I alleles among the study population diagnosed during primary HIV infection [PHI].

| HLA-A | Symptomatic PHI (n=68) | | p | Progressor at one year (n=58) | | p | All (N=68) |
| --- | --- | --- | --- | --- | --- | --- | --- |
|  | Yes (N=53) | No (N=15) |  | Yes (N=18) | No (N=40) |  |  |
| A*01 | 3 (2.8) | 1 (3.3) | 1.00 | 0 | 4 (5) | 0.415 | 4 (2.9) |
| A*02 | 26 (24.5) | 11 (36.7) | 0.245 | 10 (27.8) | 21 (26.2) | 1.00 | 37 (27.2) |
| A*03 | 5 (4.7) | 3 (10) | 0.374 | 2 (5.6) | 5 (6.2) | 1.00 | 8 (5.9) |
| **A*11** | 7 (6.6) | 0 | 0.328 | **6 (16.7)** | **1 (1.2)** | **0.003** | 7 (5.1) |
| A*23 | 3 (2.8) | 1 (3.3) | 1.00 | 0 | 3 (3.7) | 0.586 | 4 (2.9) |
| A*24 | 15 (14.2) | 2 (6.7) | 0.361 | 6 (16.7) | 9 (11.2) | 0.550 | 17 (12.5) |
| A*26 | 4 (3.8) | 2 (6.7) | 0.613 | 0 | 5 (6.2) | 0.299 | 6 (4.4) |
| A*29 | 10 (9.4) | 1 (3.3) | 0.455 | 3 (8.3) | 8 (10) | 1.00 | 11 (8.1) |
| A*30 | 2 (1.9) | 0 | 0.920 | 0 | 2 (2.5) | 0.852 | 2 (1.5) |
| A*31 | 8 (7.5) | 4 (13.3) | 0.300 | 5 (13.9) | 5 (6.2) | 0.281 | 12 (8.8) |
| A*32 | 7 (6.6) | 2 (6.7) | 1.00 | 0 | 7 (8.7) | 0.159 | 9 (6.6) |
| A*33 | 6 (5.7) | 0 | 0.407 | 2 (5.6) | 2 (2.5) | 0.587 | 6 (4.4) |
| A*36 | 0 | 1 (3.3) | 0.499 | 0 | 1 (1.2) | 0.680 | 1 (0.7) |
| A*66 | 1 (0.9) | 0 | 0.499 | 0 | 1 (1.2) | 0.680 | 1 (0.7) |
| A*68 | 7 (6.6) | 2 (6.7) | 1.00 | 2 (5.6) | 5 (6.2) | 1.00 | 9 (6.6) |
| A*69 | 1 (0.9) | 0 | 0.499 | 0 | 1 (1.2) | 0.680 | 1 (0.7) |
| A*74 | 1 (0.9) | 0 | 0.499 | - | - | - | 1 (0.7) |
| HLA-B | Symptomatic PHI (n=66) | | p | Progressor at one year (n=56) | | p | All (N=66) |
|  | Yes (N=52) | No (N=14) |  | Yes (N=15) | No (N=41) |  |  |
| B*07 | 6 (5.8) | 1 (3.6) | 1.00 | 3 (10) | 3(3.7) | 0.340 | 7 (5.3) |
| B*08 | 5 (4.8) | 0 | 0.532 | 0 | 4 (4.9) | 0.511 | 5 (3.8) |
| B*13 | 1 (1) | 0 | 0.480 | 0 | 1 (1.2) | 0.598 | 1 (0.8) |
| B*14 | 9 (8.6) | 1 (3.6) | 0.688 | 4 (13.3) | 5 (6.1) | 0.246 | 10 (7.6) |
| B*15 | 6 (5.8) | 2 (7.1) | 0.677 | 2 (6.7) | 6 (7.3) | 1.00 | 8 (6.1) |
| B*18 | 8 (7.7) | 0 | 0.285 | 2 (6.7) | 5 (6.1) | 1.00 | 8 (6.1) |
| B*27 | 2 (1.9) | 0 | 0.895 | 0 | 1 (1.2) | 0.598 | 2 (1.5) |
| B*35 | 15 (14.4) | 6 (21.4) | 0.388 | 6 (20) | 12 (14.6) | 0.563 | 21 (15.6) |
| B*37 | 0 | 1 (3.6) | 0.480 | 0 | 1 (1.2) | 0.598 | 1 (0.8) |
| B*38 | 3 (2.9) | 0 | 0.845 | 1 (3.3) | 2 (2.4) | 1.00 | 3 (2.3) |
| B*39 | 3 (2.9) | 3 (10.7) | 0.109 | 1 (3.3) | 3 (3.7) | 1.00 | 6 (4.5) |
| B*40 | 7 (6.7) | 2 (7.1) | 1.00 | 1 (3.3) | 6 (7.3) | 0.672 | 9 (6.8) |
| B*41 | 4 (3.8) | 0 | 0.665 | 1 (3.3) | 2 (2.4) | 1.00 | 4 (3.0) |
| B*42 | 0 | 1 (3.6) | 0.480 | 0 | 1 (1.2) | 0.598 | 1 (0.8) |
| B*44 | 12 (11.5) | 5 (17.8) | 0.357 | 3 (10) | 11 (13.4) | 0.756 | 17 (12.9) |
| B*45 | 1 (1) | 1 (3.6) | 0.381 | 0 | 1 (1.2) | 0.598 | 2 (1.5) |
| B*48 | 2 (1.9) | 0 | 0.895 | 1 (3.3) | 1 (1.2) | 0.466 | 2 (1.5) |
| B*49 | 2 (1.9) | 1 (3.6) | 0.514 | 1 (3.3) | 1 (1.2) | 0.466 | 3 (2.3) |
| B*50 | 0 | 2 (7.1) | 0.061 | 0 | 2 (2.4) | 0.954 | 2 (1.5) |
| B*51 | 5 (4.8) | 0 | 0.532 | 1 (3.3) | 4 (4.9) | 1.00 | 5 (3.8) |
| B*52 | 1 (1) | 0 | 0.480 | 1 (3.3) | 0 | 0.598 | 1 (0.8) |
| B*53 | 2 (1.9) | 0 | 0.895 | 2 (6.7) | 0 | 0.120 | 2 (1.5) |
| B*55 | 2 (1.9) | 0 | 0.895 | 0 | 2 (2.4) | 0.954 | 2 (1.5) |
| B*56 | 1 (1) | 0 | 0.480 | 0 | 1 (1.2) | 0.598 | 1 (0.8) |
| B*57 | 4 (3.8) | 1 (3.6) | 1.00 | 0 | 3 (3.7) | 0.688 | 5 (3.8) |
| B*58 | 2 (1.9) | 1 (3.6) | 0.514 | 0 | 3 (3.7) | 0.688 | 3 (2.3) |
| B*83 | 1 (1) | 0 | 0.480 | 0 | 1 (1.2) | 0.598 | 1 (0.8) |
| **HLA-C** | Symptomatic PHI (n=68) | | p | Progressor at one year (n=58) | | p | All (N=68) |
|  | Yes (N=53) | No (N=15) |  | Yes (N=18) | No (N=40) |  |  |
| C*01 | 4 (3.8) | 0 | 0.640 | 0 | 3 (3.7) | 0.586 | 4 (2.9) |
| C*02 | 3 (2.8) | 0 | 0.820 | 1 (2.8) | 1 (1.3) | 0.526 | 3 (2.2) |
| **C*03** | 12 (11.3) | 4 (13.3) | 0.753 | **1 (2.8)** | **14 (17.5)** | **0.035** | 16 (11.8) |
| C*04 | 18 (17.0) | 4 (13.3) | 0.783 | 7 (19.4) | 12 (15) | 0.592 | 22 (16.2) |
| C*05 | 4 (3.8) | 2 (6.7) | 0.613 | 0 | 6 (7.5) | 0.217 | 6 (4.4) |
| **C*06** | **4 (3.8)** | **4 (13.3)** | **0.071** | 1 (2.8) | 5 (6.3) | 0.664 | 8 (5.9) |
| C*07 | 30 (28.3) | 8 (26.7) | 1.00 | 14 (38.9) | 19 (23.7) | 0.120 | 38 (27.9) |
| C*08 | 10 (9.4) | 1 (3.3) | 0.455 | 4 (11.1) | 6 (7.5) | 0.498 | 11 (8.1) |
| C*12 | 5 (4.7) | 1 (3.3) | 1.00 | 1 (2.8) | 4 (5) | 1.00 | 6 (4.4) |
| C*14 | 2 (1.9) | 0 | 0.919 | 0 | 1 (1.3) | 0.680 | 2 (1.5) |
| C*15 | 4 (3.8) | 1 (3.3) | 1.00 | 2 (5.6) | 3 (3.7) | 0.645 | 5 (3.7) |
| C*16 | 6 (5.7) | 4 (13.3) | 0.227 | 3 (8.3) | 5 (6.3) | 0.702 | 10 (7.4) |
| C*17 | 4 (3.8) | 0 | 0.640 | 2 (5.6) | 1 (1.3) | 0.227 | 4 (2.9) |
| C*18 | 0 | 1 (3.3) | 0.499 | 0 | 0 | - | 1 (0.7) |

*Data are no. (%) of HLA alleles.
