# Supplementary material for: Host Genetic Factors Associated with Symptomatic Primary HIV Infection and Disease Progression among Argentinean Seroconverters
Source: PLoS One. 2014 Nov 18;9(11):e113146. doi: 10.1371/journal.pone.0113146 (PMC4236131; doi:10.1371/journal.pone.0113146)
Supplement: Table S5 — Frequency of HLA class I alleles homozygosis among the study population diagnosed during primary HIV infection [PHI]. (DOC) [file pone.0113146.s006.doc]

**Table S5.** Frequency of HLA class I alleles homozygosis among the study population diagnosed during primary HIV infection [PHI].

| HLA | | Symptomatic PHI | |  | Progressor at one year | |  | All |
| --- | --- | --- | --- | --- | --- | --- | --- | --- |
|  |  | Yes | No | p | Yes | No | p |  |
| HLA-A | Homozygosis | 17/53 (32.1) | 5/15 (33.3) | 0.367 | 4/18 (22.2) | 15/40 (37.5) | 1.00 | 22/68 (32.4) |
| HLA-B | Homozygosis | 2/52 (3.8) | 0/14 | 1.00 | 1/15 (6.7) | 1/41 (2.4) | 0.468 | 2/66 (3.0) |
| HLA-C | Homozygosis | 10/53 (18.9) | 2/15 (13.3) | 1.00 | 5/18 (27.8) | 6/40 (15) | 0.290 | 12/68 (17.6) |

*Data are no. (%) of homozygosis.
